# Patients’ perception of the information security management in health centers: the role of organizational and human factors

**DOI:** 10.1186/s12911-018-0681-z

**Published:** 2018-11-15

**Authors:** Hamid Reza Peikari, Ramayah T., Mahmood Hussain Shah, May Chiun Lo

**Affiliations:** 1 Department of Management, Isfahan (Khorasgan) Branch, Islamic Azad University, Isfahan, Iran; 2 School of Management, Universiti Sains Malaysia, Penang, Malaysia; 3 School of Strategy and Leadership, Coventry University, Coventry, UK; 4 Research & Innovation Management Centre (UNIMAS INNOVATION), Universiti Malaysia Sarawak (UNIMAS), 94300 Kota Samarahan, Sarawak Malaysia

**Keywords:** Security, Trust, Technical and physical protection, Monitoring, Training, Ethics

## Abstract

**Background:**

Researchers paid little attention to understanding the association of organizational and human factors with patients’ perceived security in the context of health organizations. This study aims to address numerous gaps in this context. Patients’ perceptions about employees’ training on security issues, monitoring on security issues, ethics, physical & technical protection and trust in hospitals were identified as organizational and human factors.

**Methods:**

After the development of 12 hypotheses, a quantitative, cross-sectional, self-administered survey method was applied to collect data in 9 hospitals in Iran. After the collection of 382 usable questionnaires, the partial least square structural modeling was applied to examine the hypotheses and it was found that 11 hypotheses were empirically supported.

**Results:**

The results suggest that patients’ trust in hospitals can significantly predict their perceived security but no significant associations were found between patients’ physical protection mechanisms in the hospital and their perceived information security in a hospital. We also found that patients’ perceptions about the physical protection mechanism of a hospital can significantly predict their trust in hospitals which is a novel finding by this research.

**Conclusions:**

The findings imply that hospitals should formulate policies to improve patients’ perception about such factors, which ultimately lead to their perceived security.

## Background

Information security breaches result in an average of $7 Billion worth of losses every year in the healthcare industry [1]. This has motivated many researchers to conduct research from different perspectives with an aim to reduce the likelihood of security breaches and the costs associated with it. While the researchers in the field of computer science have explored the phenomenon of information security, most of them have studied the issue from the engineering perspective and focused on the development of technical solutions and neglected to study security from a behavioral approach [2]. Behavioral approach refers to studying the factors which shape individuals’ perception and behavior towards the practice of information security in organizations. Therefore, this research intends to answer the following question: what are the anticipating factors of individuals’ perceived security?

Investigating the antecedents of information security from the behavioral perspective is important because it plays an important role in evaluating and ensuring the level of information security. Indeed, since most individuals lack technical knowledge of the security technologies implemented, they assess the data protection levels of the organization based on some cues. This phenomenon is called perceived security. Therefore, in order to reduce individuals’ concerns about the security of their information, the factors that play a significant role in shaping their perceived security should be enhanced.

Although some studies related to behavioral security have been conducted, there are some gaps associated with such research. Some studies in this field, have focused on perceived security in online environment [3–8], and have emphasized only the role of technical factors and ignored the role of organizational and human factors in influencing individuals’ perceived security. Organizational factors in this context refer to the managerial and organizational policies practiced (such as monitoring and training the employees, or the deployment of technical and physical protection equipment) to address the information security issues in the organization. Human factors, on the other hand, refer to individual employees’ behaviors and practices (such as employees’ ethics), which can strengthen or weaken information security situations in the organization. The lack of sufficient research in this field has led to the emergence of a new research stream, which has highlighted the role of organizational and human factors, besides the technical factors, in studying perceived security [1, 8–12].

While most of the past research on the perceived security is in the field of e-commerce or employees’ perceived security, studying the factors that enhance patients’ perceived security (in the field of health information) is crucial. Over time, a patients’ medical and health record accumulates sensitive individual information, which may be misused by unauthorized parties [9]. This makes patients concerned with regards to the potential unauthorized disclosure and misuse of their information. Patients have to provide information to the health service providers to help them better diagnose and prescribe, leading to facilitating the provision of healthcare services. However, patients may refuse to share sensitive, private and important information where there are potentially embarrassing health problems such as HIV or psychological disorders, due to their concern regarding the disclosure of such information to non-authorized parties and people [9]. This is because they may feel that disclosure will result in social shame and discrimination. Consequently, such concerns and non-disclosure of sensitive information may worsen the patients’ health conditions, exposing their lives to risk. Hence, studying the factors which contribute in the patients’ perceived security of their sensitive information is an important factor for ultimately providing effective health services to them. Despite the importance of the issue, limited studies have focused on health sector, which calls upon models different from other sectors [9]. The violation of patients’ information security is the second highest reported breach [9], implying that patients are concerned of such violations and threats. Appendix illustrates the factors and contexts studied in the past research related to the behavioral security. As shown, all the papers illustrated in Appendix have focused on employees’ perceptions and the factors preventing employees from a security breach in organizations and none of them have examined the factors leading to behavioral information security in the context of health sector and from the view point of patients. In other words, the models and findings presented by the past research cannot be applied to the context of health information security from the patients’ perspective and this field lacks a thorough understanding of what makes patients perceive that their information is protected against security threats.

Another research gap in the context of perceived information security is regarding the antecedents of perceived security in the past research. As shown in Appendix, most of the past research has examined perceived certainty and severity of penalties (sanctions/penalties), normative belief, attitude and self-efficacy. Most of the past studies in this field have used general deterrence theory to study the antecedents of perceived security. However, more factors other than those examined in the past research have been suggested as the antecedents of perceived security by some researchers. For instance, perceived employees’ training [1, 2, 11, 13–16], monitoring [11, 15, 17], physical and technical protection [18, 19], and ethics [15, 17, 20–22]. The researchers however neglected to empirically examine the association of the mentioned factors with perceived security. Hence, it is essential to study the association of such factors in the model. Moreover, the dominant theory in behavioral security studies is the general deterrence theory, which has made our knowledge and understanding limited and hence, more theories should be used in this field to enrich the knowledge available in this field.

Considering the above research gaps, this research aims to develop and validate a model which predicts patients’ perceived information security. Therefore, as discussed earlier, six factors, namely technical protections, physical protection, trust in hospital, monitoring employees, security training and security were identified as the less-studied factors in the past research. Therefore, the mentioned factors were considered as the potential antecedent of perceived security and this research intends to examine their relation with patient perceived security. The findings and implications of this paper will contribute in the academic front by posing and examining a new theoretical model to understand the interrelations that exist between the determinants of patients’ perceived security. This can enrich the existing theories and knowledge regarding the determinant factors of individuals’ perceived security. The findings can also help managers and practitioners in the healthcare industry gain a better knowledge and understanding of the patients’ perceived security which in turn enables them to provide effective and efficient provisions designed to address and improve patients’ perceived security. This can lead to the patients’ disclosure of critical, sensitive information which ultimately helps improve the delivery of higher quality health services to the patients.

### Literature review and hypotheses development

Chellappa and Pavlou [3] refer to perceived security as the individuals’ belief of the subjective probability that their sensitive information will not be accessed, by inappropriate parties, and in accordance with their confident expectations. According to [15], security has an impact on organizational technology, processes and the employees’ manner in processing information. While some researchers have studied the role of technical factors and solutions in the protection of information security [4, 6, 8], some others have highlighted the role of human factors with regards to information security threats in organizations [1, 9–11, 13]. Hence, both factors should be considered in the evaluation of information security in organizations.

According to [10], health organizations’ success in protecting information security is rooted in two factors: i-technical aspects and ii-organizational and human factors. Therefore, this research categorizes threats to patients’ information security into two main areas:

1. Technical threats, rooted in the technical vulnerabilities of the information systems; and
2. Organizational and human threats, rooted in inappropriate/unauthorized access of patients’ information by internal parties, abusing their privileges.

The first form of threat can be managed by utilizing robust technical solutions to deal with the technical threats to penetrate the system and access sensitive data with no authorization. On the other hand, the second type of threat can be managed by organizational policies such as training the personnel to protect sensitive data, monitoring them to make sure they do not violate any rules and communicating the principles of ethics amongst personnel to encourage ethical working practices. Hence, in this research, the technical and physical protection variables are considered as the technical aspect of information security while employees’ training, ethics and monitoring are considered as the organizational determinants of perceived security. Moreover, since there is an association between trust and security [23, 24], we propose and consider trust in hospital as an antecedent of security.

This research refers to cue utilization theory, cue consistency theory and environmental psychology to develop its theoretical foundation. According to cue utilization theory, the quality of a product or service can be assessed by two different cues: (1) extrinsic cues, and (2) intrinsic cues. The former refers to alterable product/service attributes and the latter are related to non-alterable, inherent product/service features and characteristics [8, 25]. This holds true for information security protection as a service to be offered and ensured for patients in hospitals. When patients refer to a hospital, they evaluate the services offered by the hospital by using intrinsic cues; and extrinsic cues such as security policy, monitoring, training, or operating policy statements of the hospital. When numerous cues are consistent, a synergic interaction is created among them and the presence of each cue strengthens the association of the other cues, which is called the Cue Consistency Theory [25]. Therefore, patients use their perception regarding different factors and evidences as cues to make judgments about the unknown [26]. According to the environmental psychology, a place’s atmosphere can influence individuals’ beliefs about that place [8]. Therefore, if an organization (including a hospital) has an atmosphere to assist clients to find it trustworthy, they will perceive it as a reliable organization that does not intend to violate its clients’ interests, including the security of their information [27]. Clients may look for organizational factors and features to judge the confidentiality measures and security of their information [28]. Moreover, a well-managed organization might influence clients’ perceptions that their information will be safe and secured [8]. Hence, organizational factors can predict perceptions regarding the security of information.

### Physical protection, employees’ monitoring, and training lead to security

Colwill [13] argues that employees’ training is the greatest non-technical tool to protect the information security in organizations. Health organizations do not usually employ security trained staff, which leads to vulnerabilities in their information security [1]. Training staff to improve their knowledge and awareness on security issues and threats is one of the best non-technical solutions, which prevents insiders from disclosing the sensitive information to unauthorized parties [11, 13]. Good training and effective and efficient policies to deal with security threats are good sources of preventing security breaches in health organizations [1, 14, 16]. Training can increase staff knowledge and awareness about the threats and consequences of a security breach, leading to the prevention of such incidents [21]. Likewise, [15] speculate that employees’ training and monitoring can influence the security of information in organizations. Personnel monitoring is used by organizations to ensure that their employees adhere to their rules and regulations. According to [11], employee monitoring and surveillance reduces the likelihood of an employee related security breach by increasing their perceived certainty and severity of punishments and the potential consequences for such behaviors. According to [17], monitoring employees to find and correct their unacceptable behavior can lead to the deterrence of problematic behaviors, including security breaches. Monitored employees are very unlikely to take risks with regards to disclosing sensitive information and take care of their responsibilities in relation to information security. Hence, it is suggested that:

1. Patients’ perceptions about the training of employees on information security have a positive relationship with their overall perceived information security.
2. Patients’ perceptions about the monitoring of employees on information security have a positive relationship with their overall perceived information security.

Moreover, physical protection aids information security by deploying measures that are too difficult to defeat [19]. Physical protection is the third stream of security management in conjunction with policies and personnel countermeasures [18]. A physical protection mechanism integrates procedures, people, and tools to protect the assets against sabotage, theft, and terrorist attacks [19]. Indeed, when an intruder intends to access the information, one way is to personally and physically access the data storage/transition instruments. Hence, when an organization deploys robust physical protection mechanisms (such as locks, CCTV, etc), the intruders cannot easily access the systems and hence the likelihood of security breach is reduced. So, when patients observe robust physical protection measures, they perceive that the security of their information is protected. Hence, it can be hypothesized that:

H3. Patients’ perceptions toward the robustness of physical protection mechanisms have a positive relationship with their perceived information security.

### Physical protection, training, monitoring, technical protection and ethics lead to trust

There are two different types of trust: trust in technology and trust in the service/goods provider [24]. This research refers to trust as the later form. In this sense, trust is an attitude of confidence towards a party [29]. According to [30], one of the prerequisites of trust in an organization is perceived ethics (also called benevolence), which deals with the perception that the trustee cares about the benefits of the trusting party to protect the rights of the trusting party. Indeed, ethics refer to the belief about the goodwill of another party. Perceived ethics reduces perceived uncertainty by making the trusting party to ignore the trustee’s undesirable behavior. According to [31], an ethical party tries to adjust to the trusting party needs. This can be achieved by the trusted party’s observation and application of rules, procedures and policies to ensure the benefits of the trusting party. Johnson [32] illustrates that customers’ belief in the ethical practices in an organization positively influence their trust in the organization. Indeed, individuals’ perceptions about the extent of which a service provider adheres to ethical values and codes of ethics indicates the extent to which the service provider is willing to tolerate opportunistic behavior. Therefore, the more the service provider is perceived as an ethical party, the less would be the likelihood of unfair practices in the interaction, which reduces the perceived level of transaction uncertainty and risk. Some researchers have associated perceived ethics of the organization with individuals’ trust in it [33, 34]. Hence, it can be suggested that:

H4. Patients’ perceptions about the ethical practices in a hospital have a positive relationship with their trust in the hospital.

Belanger et al. [35] on the other hand have referred to institutional, structural-based trust as the belief that trust is likely because of regulations, promises, guarantees, legal recourse, contracts, processes or procedures. Likewise, [23] uses institutional-based trust as the belief of a trustor about the security of a situation based on the guaranteed safety procedures, policies and practices. Physical protection, training, monitoring, technical protection and ethics can be considered as the key factors in shaping patients’ trust. Technical protection are the overall technical solutions and capabilities deployed by the information technology department to ensure the confidentiality of the transmitted information [8]. For instance, [5] found that perceived technical protection in the context of e-payment systems can significantly predict their trust in the system. Likewise, [6] found that technical protection can significantly predict customers’ trust in the e-commerce context. According to [36], in the e-commerce context, customers’ perceived technological trustworthiness of a website enhances their trust in the website. They maintain that the lack of technical reliability can end in users’ loss of trust. In another research, [8] speculate that the overall technical capabilities of an organization to ensure the security of the exchanged information can lead to individuals’ trust. Likewise, when an organization formulates and implements certain policies, such as the provision of training to its staff, deploying physical protection to deter a security breach and monitoring the staff to prevent their abuse, this can lead to individuals’ trust in the service provider. It is in line with [29] who refer to trust as the attitude of confidence towards a party. It is applicable in the context of the health sector because patients believe that the hospital does not try to violate their rights and endanger them by improper policies and practices. Hence, it is suggested that:

H5. Patients’ perceptions toward the physical protection capabilities has positive relationship with their trust in hospitals.
H6. Patients’ perceptions toward the technical protection capabilities has positive relationship with their trust in hospitals.
H7. Patients’ perceptions toward the staff training on security issues has positive relationship with their trust in hospitals.
H8. Patients’ perceptions toward staff monitoring has positive relationship with their trust in hospitals.

### Ethics leads to security

Organizations should build an effective culture among their employees to ensure data security [15]. Ruighaver et al. [17] speculate that organizations should encourage ethics in situations where information security is at risk. Adherence to ethics can become a culture amongst employees, which leads to the protection of data [15]. The ethical principles have been developed for health professionals to encourage them to take on responsibility of protecting information security [21]. Many of the security and privacy threats could be prevented if the computer users observed the ethical standards in the other interacting parties [20, 22]. D'Arcy et al. [11] suggest employees’ ethical behavior as an important prerequisite for information security. Likewise, [13] state that employees may threaten the security of the information systems due to the lack of ethics. Therefore, it is suggested that:

H9. Patients’ perceptions toward the ethical practices in a hospital has positive relationship with their perceived information security in the hospital.

### Training leads to ethics

According to [22], organizations should provide training to their employees to promote their ethical practices. They speculate that many of the ethical violations could be prevented by training the employees. Employees’ training programs can include the organization’s expected code of conduct and ethics [37]. When the employees are made aware that their organization rewards good behavior and adherence to ethics, they are more likely to adhere to the ethical guidelines of the organization [13]. According to [19], training employees can form and enhance an ethical culture in the organization to influence personnel to act ethically and feel responsible for the protection of information. Hence, it is suggested that:

H10. Patients’ perceptions about the employees’ training has positive relationship with their perceptions about employees’ ethics in the hospital.

### Trust leads to security

While some researchers have found that individuals’ perceptions about the security and privacy features of a technology lead to their trust in the technology [23, 24, 31, 38], others have found that individuals’ trust is one of the antecedents of their perceived risk and security in online environment [27, 29, 39, 40, 41]. Likewise, [42] found that individuals’ trust in the context of e-commerce could negatively influence their perceived risk. The higher the degree of trust, the lower the degree of uncertainty and perceived risk by customers [43]. This is because trust in an organization can reduce individuals’ uncertainties in dealing with the organization. In other words, when individuals can trust in an organization, they perceive fewer risks in their relationship and interactions with the organization. This includes the risk on information security. Hence, it is hypothesized that:

H11. Patients’ trust in a hospital has a positive relationship with their perceived information security in the hospital.

### Technical protection leads to perceived security

Kim [5] argue that an acceptable level of data integrity and stability can enhance customers’ perceived security in the e-payment context. Since it is difficult for individuals to assess the technical protection robustness from the technical perspective, they evaluate it based on their perceptions on the functionality of these mechanisms [6, 8]. Hence, this research also utilizes this approach to evaluate technical protection mechanisms in hospitals. In the context of e-commerce, [6, 8] found that customers’ perceived technical protection can predict their perceived security. Hence, it is suggested that:

H12. Patients’ perceived technical protection capabilities of the hospitals has positive relationship with their perceived information security.

## Methods

This study used a quantitative, self-administered survey method and collected data by using a cross-sectional approach. The questionnaire was composed of 38 questions, which was expected to take less than 15 min on average for respondents to be filled out. As shown in Table 1, the questions were adopted and adapted from other sources. Apart from the demographic questions, the other questions used a 5-point Likert scale. Before collecting the data, the questionnaire was independently checked by three academics and three practitioners who were experts in the field of information security and the scale was revised according to their comments. This indicates the face and content validity of the scale. The questionnaire was then tested in a pre-test stage, with five respondents, testing the questionnaire separately.

**Table 1:** Questionnaire Details

Variable	No. of Items	Sources	Reliability
Perceived Physical Protection	7	[71]	0.82
Perceived Security	3	[72]	0.89
Perceived Ethical Practice	6	[73–75]	0.87
Perceived Monitoring	7	[76, 77]	0.84
Perceived Training	5	[78]	0.85
Trust in Hospital	3	[39]	0.75
Technical Protection	4	[72]	0.82

After the process of content validity at the pilot study stage, a convenient sampling method was utilized. The target population was consisted of the patients of 9 educational hospitals in Isfahan. Since the population size was greater than 100,000 people; the sample size was 384 respondents, following the sample size table outlined by [44]. To meet this number, 450 questionnaires were distributed amongst the patients of the mentioned hospitals. The participation in the data collection process was voluntarily and the participants were ensured that their identification and answers will be kept confidential. After 1 month, 382 usable questionnaires were collected, which is very close to the 384 sample size outlined by [44] for large populations. One probable reason for such a high response rate was that the patients had sufficient time to complete the questionnaires between the time they had entered the hospital and the time they were admitted. However, we had a small portion of lost or incomplete questionnaires. After the collection of the questionnaires, descriptive statistics was carried out by SPSS 20; while partial least square (PLS) modeling technique, using SmartPLS 3.0 was utilized to assess the construct validity and examine the hypotheses.

## Results

### Demographic results

As shown in Table 2, most of the respondents were older than 50 years old (45.29%), followed by those between 41 and 50 years old (21.2%). Moreover, the analysis revealed that there was a fairly even split between male and female respondents (52% and 47.12% respectively) and almost half of the respondents had a diploma degree (46.86%).

**Table 2:** The Results of the Demographic Analysis

Demographics	Categories	Frequency	Percent
Age (years)	Less than 21	8	2.09
	21–30	53	13.87
	31–40	67	17.55
	41–50	81	21.20
	> 50	173	45.29
Gender	Female	180	47.12
	Male	202	52.88
Education	Under diploma	43	11.26
	Diploma	179	46.86
	B.A.	43	11.26
	B.Sc.	90	23.56
	M.Sc. and above	27	7.06

### Research model analysis

To analyze the model, the Partial Least Squares (PLS) analysis technique was utilized by the SmartPLS 3.0 software [45]. Following the two-stage analytical procedure recommended by some scholars [46–50], the measurement model was tested to confirm the validity and then the structural model were tested to examine the significance of the loadings and path coefficients.

### Validity and reliability

To evaluate the measurement model in PLS, construct validity was examined by confirmatory factor analysis approach. To test the construct validity, 2 types of validity test procedures were employed; the convergent and discriminant validity. The former is usually tested by examining the path loadings, average variance extracted (AVE) and also the composite reliability [24, 51]. As shown in Table 3, the path loadings were all higher than 0.5, the composite reliability (CR) values were all greater than 0.7 and the AVE values were also all higher than 0.5. The discriminant validity of the scale was examined by following the [52] criterion. As shown in Table 4, all the values on the square root of AVE were higher than the corresponding rows, which indicates the discriminant validity. Moreover, since all the CR and Cronbach’s alpha values exceeded 0.7, it can be concluded that the questionnaire is reliable.

**Table 3:** Assessment of Measurement Model

Construct	Items	Loadings	AVE	CR
Ethics	Ethics1	0.743	0.551	0.880
	Ethics2	0.727		
	Ethics3	0.730		
	Ethics4	0.753		
	Ethics5	0.741		
	Ethics6	0.759		
Monitoring	Monitor1	0.584	0.516	0.881
	Monitor2	0.616		
	Monitor3	0.722		
	Monitor4	0.768		
	Monitor5	0.820		
	Monitor6	0.751		
	Monitor7	0.737		
Physical	Physical2	0.686	0.549	0.859
	Physical3	0.736		
	Physical4	0.790		
	Physical6	0.743		
	Physical7	0.746		
Security	Security1	0.887	0.816	0.930
	Security2	0.915		
	Security3	0.908		
Technical	Technical1	0.865	0.659	0.884
	Technical2	0.873		
	Technical3	0.859		
	Technical4	0.624		
Training	Training1	0.801	0.630	0.895
	Training2	0.813		
	Training3	0.766		
	Training4	0.768		
	Training5	0.819		
Trust	Trust1	0.871	0.731	0.890
	Trust2	0.896		
	Trust3	0.793		

Note: Item Physical1 was deleted due to low loadings

**Table 4:** Discriminant Validity Assessment

	1	2	3	4	5	6	7
1. Ethics	**0.742**						
2. Monitoring	0.639	**0.718**					
3. Perceived Security	0.559	0.566	**0.903**				
4. Physical	0.433	0.463	0.411	**0.741**			
5. Technical	0.607	0.506	0.556	0.563	**0.812**		
6. Training	0.461	0.620	0.510	0.451	0.465	**0.794**	
7. Trust	0.507	0.570	0.445	0.270	0.426	0.495	**0.855**

Note: Values on the diagonal (bolded) are square root of the AVE while the off-diagonals are correlations

### Structural model

To assess the structural model, [47, 48, 53] recommended looking at the beta, R^2^ and the corresponding t-values by using a bootstrapping procedure with a resample of 5000. They also suggested that researchers should also evaluate the predictive relevance (Q^2^).

**Fig. 1:**
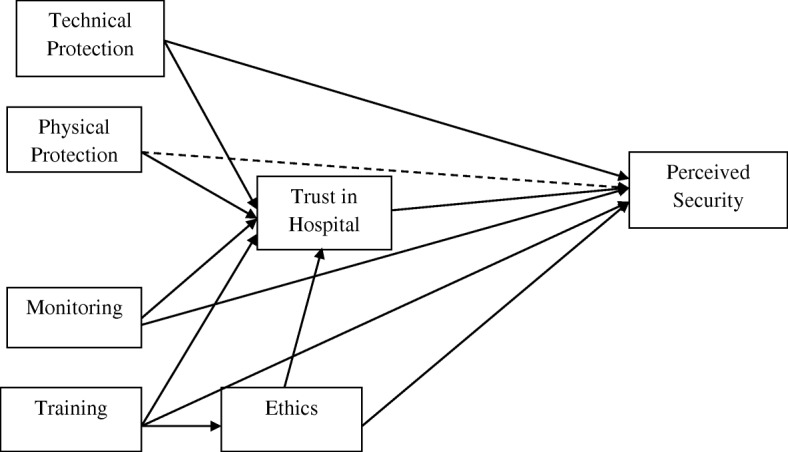
Hypotheses Results (−-->: Not Supported,➔: Supported)

First, the predictors of trust were examined and it was found that ethics (*P* < 0.01), physical protection (*P* < 0.05), technical protection (*P* < 0.05), staff training (*P* < 0.01) and staff monitoring (*P* < 0.01) had positive relationship with trust; explaining 39.8% of the variance in trust. Moreover, training had positive relationship with ethics (*P* < 0.01) with an R^2^ of 0.212. Next, the relationship of the predictors with perceived information security was examined. The results of the analyses illustrated that staff training (*P* < 0.01) and staff monitoring (*P* < 0.01), ethics (*P* < 0.01), trust (*P* < 0.1) and technical protection (*P* < 0.01) had positive relationship with perceived information security; explaining 45.7% of the variance in perceived information security while physical protection was not a significant predictor. All the R^2^ values were above the 0.35 value as outlined by [54], indicating a substantial model. The results of the hypotheses have been illustrated in Table 5 and Fig. 1.

**Table 5:** Hypotheses Testing

Hypothesis		Std Beta	Std Error	t-value	Decision
H1	Training - > Perceived Security	0.160	0.068	2.350***	Supported
H2	Monitoring - > Perceived Security	0.186	0.065	2.862***	Supported
H3	Physical protection- > Perceived Security	0.024	0.042	0.587	Not Supported
H4	Ethics - > Trust	0.187	0.059	3.176***	Supported
H5	Physical protection- > Trust	0.121	0.052	2.317**	Supported
H6	Technical - > Trust	0.123	0.058	2.107**	Supported
H7	Training - > Trust	0.212	0.057	3.693***	Supported
H8	Monitoring - > Trust	0.313	0.067	4.638***	Supported
H9	Ethics - > Perceived Security	0.181	0.064	2.845***	Supported
H10	Training - > Ethics	0.461	0.052	8.914***	Supported
H11	Trust - > Perceived Security	0.060	0.042	1.420*	Supported
H12	Technical - > Perceived Security	0.238	0.068	3.526**	Supported

***p < 0.01, **p < 0.05, *p < 0.1

In the next step, the Q^2^ was evaluated by running the blindfolding procedure in SmartPLS, which is a sample reuse procedure that deletes every d-th data point in the endogenous variable’s indicators and estimates the parameters with the remaining data points [55–57]. If the Q^2^ value is higher than 0, the model has sufficient predictive relevance for a certain endogenous variable and vice versa [48, 53, 58]. According to Hair et al. [47, 53] Q^2^ values of 0.02, 0.15, and 0.35 imply that an exogenous variable has a small, medium or large predictive relevance for a certain endogenous construct. The Q^2^ in this study was 0.282 (trust) and 0.363 (perceived security) which can be categorized as medium and large predictive relevance.

## Discussion

Security breaches impose huge financial and reputational costs to the health sector. Hence, studying the factors that can reduce security concerns are necessary. While some researchers have employed an engineering (technical) approach to measure/develop the mechanisms of security protection in organizations [59–64], others have used a subjective approach and studied the issue from a subjective perspective [3, 5, 6, 8]. Considering the importance of disclosing sensitive and vital information by patients to health staff, to receive quality and necessary health services, patients should have minimal concern about the security of their information. Therefore, this research aims to employ the subjective perspective to study the clues that patients may look for to inform their concerns on the unauthorized disclosure of their sensitive information. While some researchers have employed a subjective method to study perceived information security, they suffer from numerous shortcomings in this regard. For instance, some [4, 15, 65–69] have neglected to study the factors that predict clients’ perceptions about the security of their information in organizations and just focused on the factors preventing employees from violating the information security system and rules in organizations. Some, on the other hand, have focused on clients’ perceptions about the factors that enhance their perceived security [3–8]; however their research had only considered the technical factors and was conducted with regards to customers’ perceptions in online transactions. Hence, there were no studies which had examined the relationship of the organizational and human factors with patients’ perceived security in an Asian country. Addressing this gap was the objective of this research.

To meet the above objective, 12 hypotheses were proposed. In order to collect data, a cross-sectional, self-administrative survey was utilized and after a pilot study, 450 questionnaires were distributed amongst the patients in 9 hospitals. After 1 month, 382 usable questionnaires were collected. Since it was found that the assumption of normal distribution is violated in this research, SmartPLS 3.0 was used to analyze the hypotheses. The results revealed that apart from the third hypothesis, other hypotheses were empirically supported. The details have been illustrated in Table 5. This has numerous implications and applications.

We found that staff’s training had positive relationship with patients’ trust in hospitals (*P* < 0.01) and perceived security threats (*P* < 0.05). This is in line with some researchers who stated that staff’s training on security skills is expected to reduce the security risks in organizations [1, 11, 13, 15, 70]. This is a novel theoretical contribution in this regard, because none of the above researchers have empirically examined the relationship of patients’ perceptions about employees’ training with perceived security. The results of these two findings have numerous practical implications. First, health organizations should set policies to train their employees on information security issues such as potential threats and penetration techniques, employees’ responsibilities on protecting the security of the information, required skills to deal with security threats, legal issues, etc. Next step, in order to influence patients’ perceptions about the extent of which their sensitive information is protected, hospitals need to communicate their policies on employees’ training to their patients. This can help patients reduce their concern on the violation of their information security.

The results of the quantitative analysis found that patients’ trust in hospitals can significantly predict their perceived security at a 0.10 significance level. This is to some extent consistent with [27, 29, 39, 40, 41], who had referred to individuals’ trust as a predictor of their perceived risk and security. This is however, a novel theoretical finding of this research, because none of the above studies had empirically tested the relationship of patients’ trust with their perceived security. Moreover, these findings can contribute to hospital practices, in the sense that if a hospital’s management formulate and implement policies to enhance patients’ trust in hospital, this can ultimately lead to their perceived security. The results, however, imply that this relationship is not as significant as the association of the other mentioned predictors of perceived security.

The results of the statistical analysis found no significant relationship between patients’ physical protection mechanisms in the hospital and their perceived information security in a hospital. This is not consistent with [19], who speculated that implementing physical protection mechanisms are expected to lead to the reduction of security threats. This inconsistency can be explained from the point that although physical protection has been mentioned as one of the dimensions of information security management [18], patients may not believe that unauthorized parties can access their information physically and they may be more concern either about online security breach or insider breach.

The results, however, found that patients’ perceptions about the physical protection mechanism of a hospital can significantly predict their trust in hospitals (*P* < 0.05). This is a novel finding since the researchers found no empirical papers, which have examined the relationship of perceived physical protection mechanisms with patients’ trust in hospitals. This phenomenon can be explained by the institutional-based trust, which states that an individuals’ trust can be guaranteed by safety procedures, policies and practices [23]. Since physical protection is considered as a safety procedure with regards to information security [19], patients’ perceptions about the physical protection mechanisms can lead to their trust in hospitals. This is because the deployment of physical protection in hospital can make patients feel that the hospital cares about the benefits of its patients and hence they find the hospital trustworthy. According to this finding, hospitals should design and deploy robust physical protection mechanisms to limit unauthorized access to their information resources and then communicate such deployments to their patients. This enhances patients’ trust in hospitals, which ultimately reduces patients’ information security concerns.

The analysis also found that patients’ perceptions about the monitoring of employees has positive significant relationship with their trust in hospitals (*P* < 0.01) and security violation concerns (P < 0.01). This is in line with some researchers who stated that monitoring employees’ activities and behaviors [11, 15, 17] is expected to reduce the security risks in organizations. This is a novel theoretical contribution in this regard, because none of the past studies have empirically examined the relationship of employees’ monitoring with patients’ trust in hospital and perceived information security. While this research is one of the first, if not the only research, which has examined the relationship of perceived monitoring with patients’ trust in hospitals, this novel finding can be explained by [23]. According to [23], trust can be predicted by individuals’ perceptions on safety procedures, policies and practices. Monitoring employees can be one of the safety policies and practices in hospitals, which was found as a significant predictor of patients’ trust in hospitals. Indeed, by monitoring employees’ security behavior and actions, patients can be satisfied that the hospital tries to reduce the vulnerability in their relations with the hospital and hence, can trust in the hospital. These findings have numerous implications. Firstly, health organizations should formulate policies to monitor their employees’ security related behavior and actions by using advanced network activity monitoring systems and system usage behavior by their employees. Secondly, in order to influence patients’ trust and perceived security in hospitals, hospitals need to communicate their policies on employees’ training and monitoring to their patients. This can help patients reduce their concern on the violation of their information security.

This research also found that patients’ perceptions about staff ethics can predict their perceived information security and trust in hospitals at a 0.01 significance level. This is in line with some researchers who have speculated that patients’ perceptions about staff’s ethics can predict their perceptions about information security [13, 15, 17, 21, 22] and trust in hospitals [30, 31, 33, 34]. Moreover, this research found that patients’ perceptions about staff’s training can shape their perception in relation to the ethical behavior of staff in hospitals (*P* < 0.01); which is consistent with the past research [13, 19, 22, 37]. First, the relationship of perceived ethics with perceived security is theoretically a novel finding of this research. None of the past research in this field has empirically examined the relationship of patients’ perceived ethics with their perceived security. Second, examining the relationship of patients’ perception about staff’s training with ethics is a new theoretical contribution by this paper as all of the mentioned papers [13, 19, 22, 37] have not provided any empirical results and analysis in this regard. Third, these findings have some practical implications as well. The results imply that health organizations need to formulate some policies and programs to promote ethical culture of dealing with patients’ information amongst their employees and staff. One way to do so is by training their staff on the code of conduct and expected ethical behavior of the organization while dealing with patients’ private and sensitive information in hospitals. Moreover, they need to communicate the existence of such programs cultural values amongst their staff to the patients. This practice can ultimately enhance patients’ perceived information security in hospitals.

This research also found that patients’ perception about technical protection can significantly predict their trust in a hospital (*P* < 0.05) and perception towards information security (*P* < 0.01). These findings are in line with some researchers, who found that perceived technical protection has positive relationship with individuals’ trust [5, 36] and perceived security [5, 6, 8]. This finding, however, provides new theoretical contributions in this field. It is because none of the above research has been conducted in the context of hospital and health information, but in the context of online transactions. Hence, this research provides new insights in this regard compared to the past similar research, where the relationship of technical protection with trust and security has been examined. These findings also have some practical implications. These findings imply that hospitals should deploy robust technical mechanisms to protect patients’ information security and then communicate these solutions to their patients. This can lead to patients’ trust in hospitals and the improvement of perceived security.

## Conclusions

Overall, this research provided numerous theoretical contributions and insights. This research is one of the few attempts to examine the interrelations among the antecedents of perceived information security from the patients’ points of view, as the majority of the existing papers had either employed a technical approach to design a framework to protect information security or had concentrated on the relationship of mostly technical factors with individuals’ perceived security in the online environment. Indeed, the past attempts had numerous shortcomings: firstly, they had mostly focused on technical aspects of information security mechanisms or technical antecedents of perceived security in online environment, and secondly, they had neglected to consider and examine the relationship of organizational and human factors with perceived security in the context of health and medical organizations. Moreover, a few studies only considered organizational and human factors, but mostly measured employees’ perceptions on the predictors of their perceived security, in organizations other than health organizations. As discussed earlier, those studies which had examined organizational and human factors had neglected to examine factors such as employees’ training, monitoring, technical and physical protection, ethics and trust in hospital in their model. Hence, this research was designed and developed to address these research gaps and examine the relationship of organizational and human factors with patients’ perceived information security in hospitals.

This paper developed and empirically tested a model that contributes to the current knowledge regarding the predictors of patients’ perceived information security, which helps both practitioners and academics better understand the clues that can predict patients’ perceived information security in hospitals. The results of this study and the final model developed (as shown in Fig. 1) in this research can be used as a base-model for researchers to develop more comprehensive and complex models of patients’ perceived security in health organizations. According to the findings of this research, as illustrated in Fig. 1, the factors: technical protection, physical protection and monitoring have a positive relationship with patients’ trust; staff training is associated with both patients’ trust and ethics. Moreover, according to the empirical analyses, the factors technical protection, staff training, ethics, patients’ trust and monitoring can predict patients’ perceived security in hospital.

In terms of practical implications, hospital managers and health policy makers can have better insights to the factors which can establish and enhance perceived information security among patients to practice and communicate such values to patients.

Despite its merits, this paper is not free from limitations. First, not all the organizational and human factors have been studied in this research. For instance, factors such as employees’ commitment, culture, loyalty, or even employees’ reaction towards probable penalties they may face in the case of security breach have not been considered in this research. Moreover, another set of factors which can play an important role in predicting patients’ perceived security is patients’ psychological characteristics such as risk-taking behavior, general optimism towards information technology, etc. This research also did not consider such factors as the moderators and predictors of patients’ perceived information security. Hence, it is suggested that researchers consider these factors in developing and testing their models in future attempts. Another point, acknowledged by this research, is that this research considered patients’ trust in hospitals as a predictor of patients’ perceived security. It is suggested that future research examine the relationship of perceived security with patients’ trust in hospitals and hospital information technology. Another point is that patients’ perception of security does not reflect actual security, but it is important to reduce their concern and perceived risk of disclosure of sensitive information. Therefore, actual security of information systems in a hospital should be differentiated from patients’ perception of the security.

## Appendix

**Table 6:** Studies on the behavioural Security in Organizational Context

Study	Antecedents of Security	Dependent variable	Respondents
[79]	Mandatoriness	Precautions Taken	Employees (system Users)
[80]	Mimetic Pressure, Coercive Pressure, Normative Pressure	Level of information security control resources	Employees (system Users)
[81]	Perceived certainty and severity of sanctions, Attachment, Commitment, Involvement, Belief, Subjective Norm, Co-worker Behavior	IS security policyviolation Intention	Employees (system Users)
[11]	Perceived certainty of sanctions, PerceivedSeverity of sanctions,	IS misuse intention	Employees (system Users)
[82]	Self-Efficacy, Attitude, Normative Beliefs	Intention to resist social engineering	Employees (system Users)
[83]	Organizational Sanctions, Workgroup sanctions	intention to violate information security	Employees (system Users)
[67]	Organizational commitment, Self-Efficacy, Security Policy Attitude, Punishment Severity, Detection Certainty, Subjective Norm, Descriptive Norm.	Security Policy Compliance Intention	Employees (system Users)
[84]	Self-control	Information Security Violations	Undergraduate students
[85]	Perceived vulnerability, Perceived severity, Response efficacy, Response cost, Self-efficacy, Attitude towardcompliance with ISSP, Subjective norms	Information security compliancebehavioral intention	business managers and IS professionals
[68]	Attitude toward information security compliance, Subjective norms, self-efficacy, Locus of control	compliance behavioral intentions	Employees (system Users)
[86]	Perceived threat Severity and certainty, ResponseEfficacy, Self-Efficacy, Perceived Cost, Vendor support, IT Budget, Firm Size	Intention to Adopt Anti-malware by SME Executives	Information systems experts
[87]	Perceived susceptibility, Perceived severity, Perceived benefits, Perceived barriers, Cues to action, General security orientation, Self-efficacy,	Computer security behavior	Employees (system Users)
[88]	Conservative Behavior, Exposure to Offence, Risk Perception	IS users’ risky behaviors threatening information security	IS users’
[89]	Self-Efficacy in Information Security	Security Practice Technology, Intention to Strengthen Security Effort, Security Practice Care Behavior	Business management students
[90]	Severity, Vulnerability, Response efficacy, Self-efficacy, Attitude, Normative beliefs, Rewards	Intention to comply with information security policies	Employees (system Users)
[91]	Intention, Trust, Organization Support,	Information security knowledge sharing	Employees (system Users)
[92]	Attitude, subjective norm, behavioral control, Threat appraisal, Self-Efficacy	Information security conscious care behavior	Employees (system Users)
[93]	Information security knowledge sharing, Information security collaboration, Information security intervention, Information security experience, Attachment, Commitment, Personal norms, Attitude	attitude towards compliance with information security policy compliance	Employees (system Users)
[94]	Perceived severity, Perceived vulnerability, Response efficacy, Self-efficacy, Perceived realism, Response cost, Rewards	Intention to IS security compliance	Employees (system Users)
[16]	Data Evaluation, Risk Analysis, Training, Integration, Policies, Legislation/Regulation, Architecture	Human Resource IS security	No data collection/No respondents

## Abbreviations

AVE: Average variance extracted
CR: Composite reliability
PLS: Partial Least Squares
Q^2^: Predictive relevance
